# Functional analysis of sandstone ground stone tools: arguments for a qualitative and quantitative synergetic approach

**DOI:** 10.1038/s41598-020-72276-0

**Published:** 2020-09-25

**Authors:** Andrea Zupancich, Emanuela Cristiani

**Affiliations:** DANTE Diet and Ancient Technology Laboratory, Department of Oral and Maxillo Facial Sciences, Sapienza University of Rome, Via Caserta 6, 00161 Rome, Italy

**Keywords:** Archaeology, Archaeology

## Abstract

In the last few years, the application of quantitative methods in the field of use wear analysis has grown considerably, involving the use of different techniques. A development in surface measurements approaches has become necessary as standard assessments based upon qualitative functional analysis are often affected by a degree of subjectivity and a limited reproducibility. To advance the current methodological debate on functional analysis of ground stone technology, we present a combined methodological approach, including qualitative and quantitative analyses, applied to the study of experimental sandstone ground stone tools. We test surface quantification at a macro and micro-scale, paired with the observation and description of residue and use wear connected to the processing of plant, animal and mineral matters. Our results provide an exhaustive quantitative dataset concerning surface modifications associated with different uses and suggest an analytical workflow for the functional analysis of both experimental and archaeological ground stone assemblages. We also highlight the limitation and pitfalls of an exclusive adoption of quantitative methods in the study of ancient tool use demonstrating how a synergetic approach can enhance the quality, reproducibility and comparability of functional data.

## Introduction

Understanding the use of ancient tools through the study of macro and micro traces represents a valuable means to investigate and reconstruct past human biographies. Since the pioneering work of S.A. Semenov^1^, the field of use wear and residue analysis has grown considerably, in terms of equipment and methodological frameworks, leading to highly reliable functional interpretations. However, the researcher’s perspective may influence the way traces are described and analysed, hence hampering objective functional interpretations. In particular, a low degree of accuracy in blind tests has been pointed (see^2,3^) together with the need to implement quantitative approaches to obtain more accurate use wear datasets and guarantee the comparability and reproducibility of functional results^3,4^. In this regard, several works have discussed the application of quantitative methods focused on surface measurements to knapped and ground stone tools (henceforth GSTs), at both macro and micro-scale^5–14^. A variety of equipment and techniques has been tested, including confocal microscopy^5,6,9,15^, atomic force microscopy^16^, surface textural imaging^17^, laser profilometry^9,18^ and 3D modelling^11–13,14,19,20^. These works paved the way to a new era of functional studies where the objectivity and reliability of functional interpretation are perceived as counterposed against well-established qualitative use wear approaches.

Amongst stone tools, GSTs are probably best suited for testing the potential of quantitative techniques in the analysis of use-related surface modifications. Not only they possess an evolutionary and behavioural significance (their use is recorded since the earliest stages of human history, throughout prehistoric times as well as within extant non-human primates^14,21–23^) but they are also characterized by long-life cycle and potential for multiple uses/used areas. Detailed functional analyses have been carried out on prehistoric GST technology, which shed light on the involvement of macro-tools in a plethora of daily life activities, ranging from food grinding and pounding to pottery burnishing, metal working, wall sanding, etc.^24–29^. However, despite the long history of research on prehistoric GSTs, we believe there are important issues that still need to be addressed in order to advance the reliability of functional interpretations concerning this technology: (a) the demand for a shared analytical workflow for the functional analysis of both experimental and archaeological GST assemblages; (b) the lack of an exhaustive quantitative dataset concerning surface modifications associated with different uses; (c) to what degree residues hinder the development and appearance of use wear through optical microscopy; (d) the need to consider GSTs in their entire dimensionality^11^; and (e) the role of the researcher subjectivity in the functional interpretation.

In this paper, we address the abovementioned points by applying a multi-level analytical approach combining both qualitative and quantitative techniques to 9 experimental sandstone GSTs. Our experimental sample was used to monitor patterns of surface modification occurring in various activities and gestures involving animal, vegetal as well as mineral matters. We performed a quantitative analysis of surface modifications at a macro and micro-scale, in combination with the qualitative description of residue and macro wear. We also investigated the role of residue adhering to the used surfaces and its impact on surface measurements and appearance. Our results indicate that, while the application of synergetic approaches is still rare^11,12,14,19,20,23,30^, the combination of qualitative and quantitative analyses is likely to provide accurate and reliable functional interpretations where the researcher’s subjectivity has an active role. We also offer a preliminary quantitative functional dataset associated with the use of sandstone GSTs and discuss the interpretative potential of specific variables for assessing different materials. We expect our results to contribute to the development of the field of functional study of GST  use, in particular, identifying the pros and cons of the application of quantitative analysis.

## Results

A sample of 9 experimental sandstone GSTs, including both active (no.2) and passive (no.7) elements, was analyzed (Supplementary Table 1). Rounded sandstone pebbles selected as GST replicas were collected from the bed of Bojetinska River in the Danube Gorges (Central Balkans, Serbia), during summer fieldwork in 2018 and 2019, within the scopes of the HIDDEN FOODS—ERC Starting Grant Project. Our selection was driven by specific methodological needs, such as (1) establishing a clear analytical workflow for the functional analysis of GSTs; (2) implementing qualitative and quantitative dataset of the surface modifications associated with different GSTs’ utilizations; and, (3) building up a specific reference collection for interpreting ancient functional biographies of GSTs recovered at various Mesolithic sites of the Danube Gorges.

The raw material selected for the experimental trials was homogeneous and characterized by grains with a high degree of angularity, densely distributed within the matrix, and  with sizes varying between 0.2 and 1 mm. The experiments performed with the replicas included the processing of animal, plant and mixed animal and mineral matters. The range of gestures performed varied according to the worked substance and comprised thrusting (no. 2), resting (no. 6) and mixed (no. 1) percussion. Each tool was used for a single activity exception made for one experimental replica (SrM-8) for which two available surfaces were used to process wild grass grains (*Aegilops ventricosa*—SrM-8a) and tendons (SrM-8b).

### 360° Surface morphometrics

Surface depressions and roughness were measured over the entire tool, allowing for the identification of specific spatial distribution patterns of the utilized areas.

#### Animal matters (Fig. 1a–e,j–n)

Differences in the distribution of low/high roughness areas are recorded. In the case of the base used to split metapodials (SrM-7), no specific low roughness areas are visible across the utilized surface as observed on the tools used to abrade bone (SrM-13) and pound tendons (SrM-6b). Low roughness areas are, on the contrary, well distributed across the surface of the tool used for cleaning fresh hide (SrM-9), while an area of high roughness characterizes the lower portion of the tool utilized to soften dry hide (SrM-6). The highest depth (mean − 0.09 mm) in surface depressions is measured on the base used to split metapodials (SrM-7), while the shallowest depressions (mean − 0.004 mm) are recorded across the utilized areas of the tools used to soften dry hide (SrM-6) and abrade dry bone (SrM-13).

**Figure 1:**
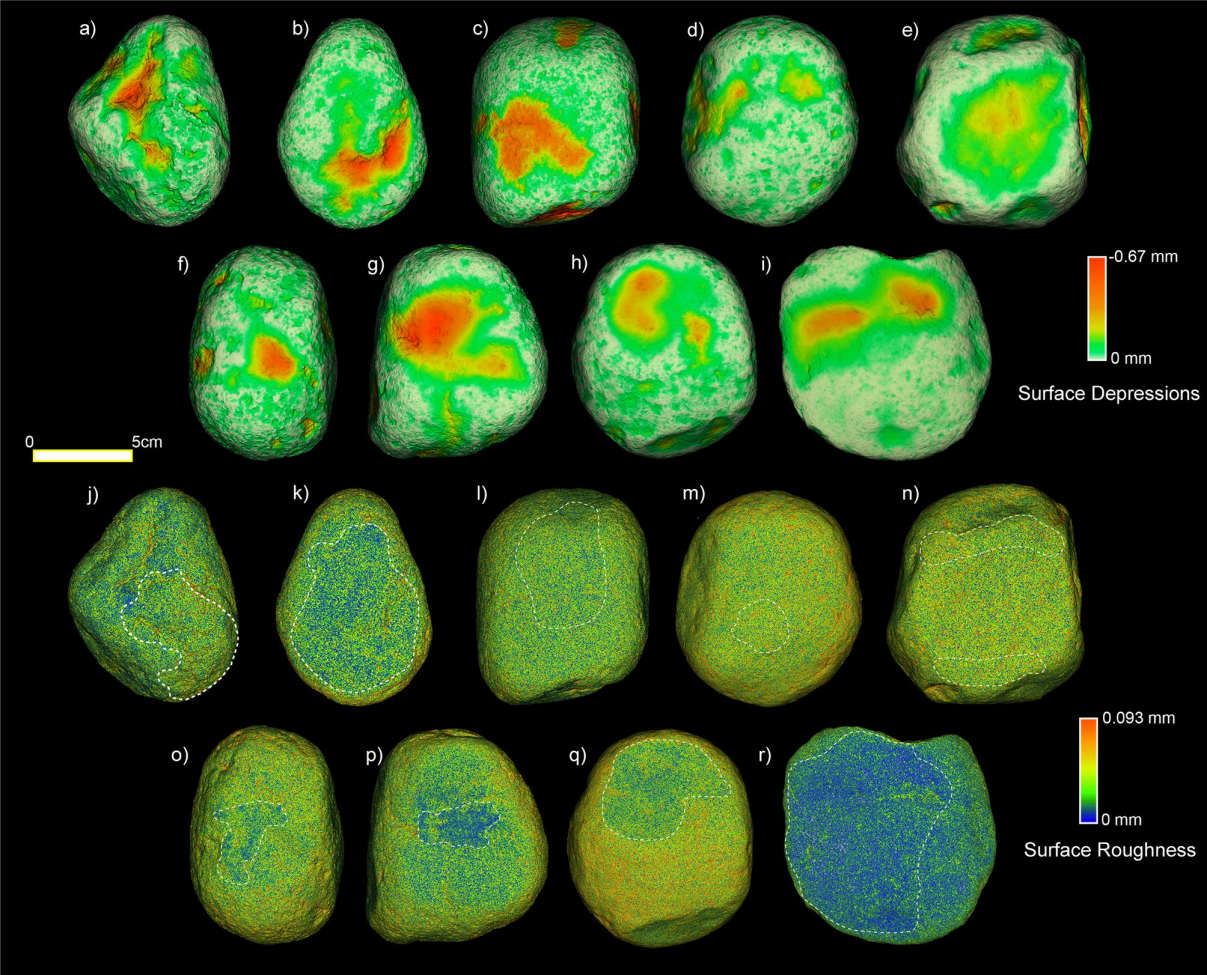
Analysis of surface depressions and surface roughness through 3D 360° surface morphometric analysis. (**a**–**e**,**j**–**n**) GST replicas utilized to process animal matters. (**a**,**j**) softening dry hide; (**b**,**k**) cleaning fresh hide; (**c**,**l**) abrading bone; (**d**,**m**) pounding tendons; (**e**,**n**) splitting metapodials. (**f**,**o**) GST replica utilized to mix ochre and marrow. (**g**–**i**,**p**–**r**) GST replicas utilized to process plants. (**g**,**p**) grinding oat; (**h**,**q**) grinding wild grass grains; (**i**,**r**) crushing and grinding acorns. White dashed line indicates the utilized area(s) of the surface.

In terms of surface roughness, the highest value (mean 0.023 mm) is recorded for softening dry hide (SrM-6) while the lowest value (mean 0.018 mm) characterizes the surfaces of tools utilized in splitting metapodials and spreading ochre over fresh hide (SrM-7 and SrM-9) (Supplementary Table 2).

#### Mixed organic and inorganic matters (Marrow and Ochre) (Fig. 1f,o)

Concerning the distribution of low roughness areas, the stone base used to mix ochre and marrow exhibits distinct patches of low roughness (mean 0.016 mm) across the utilized surface (SrM-5). Surface depressions across the utilized surface returned a mean depth of − 0.7 mm (Supplementary Table 3).

#### Vegetal matters (Fig. 1g–i,p–r)

Clear differences in the distribution of low roughness areas are recorded in the tools used to process acorns, wild grass grains and oat. In particular, areas of low roughness are distributed across the utilized surface on the tools used to process acorn and wild grass grains (SrM-16 and SrM-8), while low roughness areas appear concentrated at the centre of the tool selected for oat grinding (SrM-17).

The deepest surface depressions are associated with acorn crushing and grinding (mean − 1.5 mm) (SrM-16), while the shallowest surface depressions (mean − 0.4 mm) are recorded on the experimental replica utilized to grind wild grass grains (SrM-8). This latter also returned the most homogeneous surface, with a recorded roughness mean value of 0.011 mm while a higher roughness (mean 0.016 mm) is exhibited by the tool utilized to process acorns (SrM-16) (Supplementary Table 4).

### Residue, use wear analyses and micro surface quantification

Different patterns of residue distribution and appearance have been recorded at a micro-scale (10×–100×) along with changes in the surface topography both from a qualitative and quantitative point of view.

#### Animal matters

**Figure 2:**
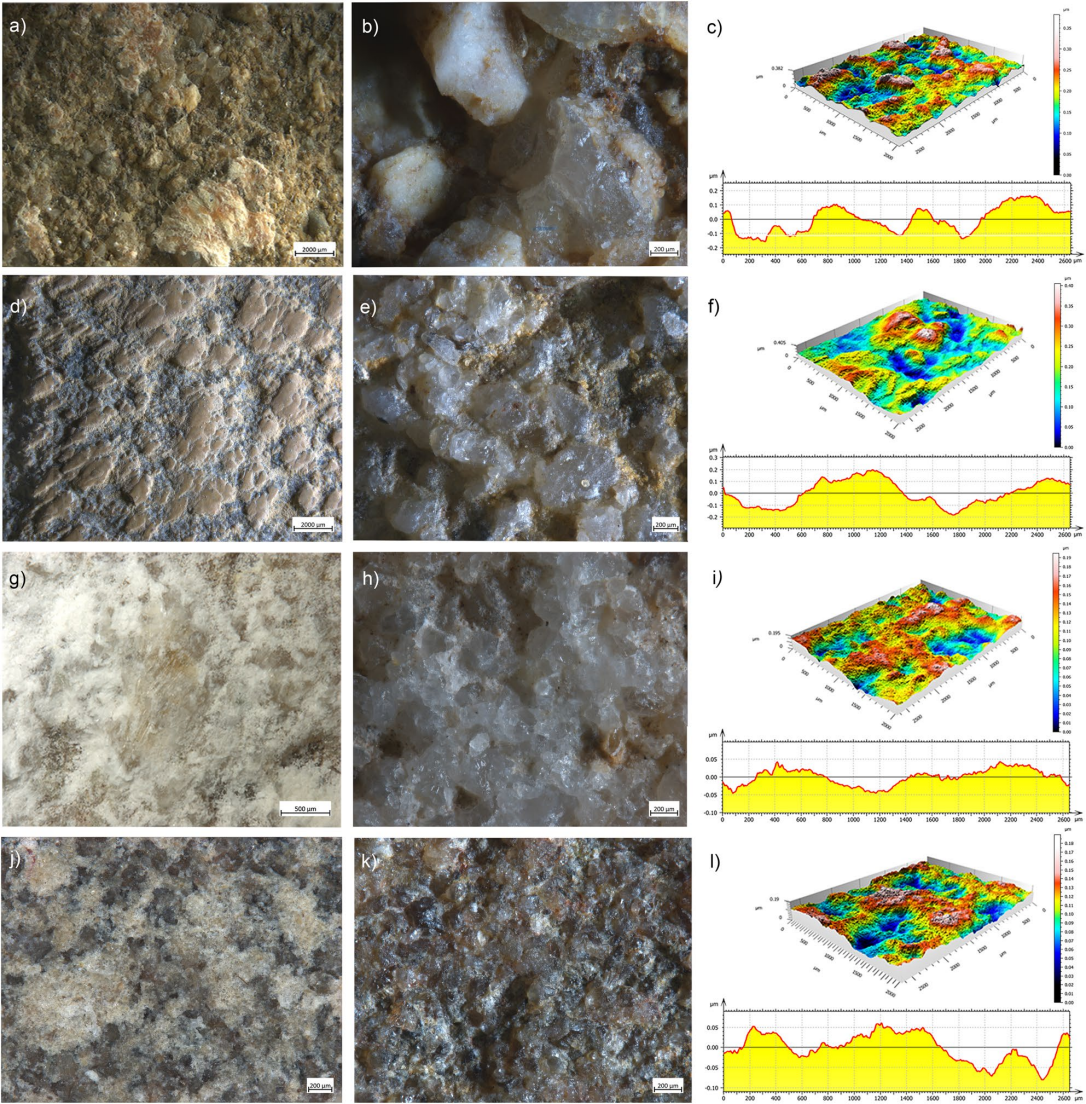
Use wear and residues observed on the experimental GST surfaces. (**a**) residues deriving from softening dry hide; (**b**) use wear observed at 50×; (**c**) 3D rendering and profile of an area of the utilized surface; (**d**) residues deriving from the cleaning of fresh hide with ochre; (**e**) use wear observed at 50×; (**f**) 3D rendering and profile of an area of the utilized surface; (**g**) residues deriving from abrading dry bone; (**h**) use wear observed at 50×; (**i**) 3D rendering and profile of an area of the utilized surface; (**j**) residues from pounding tendons; (**k**) use wear observed at 50×; (**l**) 3D rendering and profile of an area of the utilized surface.

Dry hide softening left patches of compressed whitish collagen fibres sometimes covered by an organic translucent film. The latter film appears as a thick crust distributed over the higher surface points or can merge with the lower areas of the stone matrix (Fig. 2a). In the former case, possibly due to the prolonged friction during the use, such film appears compact, translucent, dark in colour, and striated.

Observed at 50× of magnification, the microrelief of the utilized surface appears rounded. Crystal grains are clearly distinct from each other. Both the edges of the grains and the surfaces are affected by the use and appear highly rounded (edges) and abraded (surfaces) (Fig. 2b,c). Pits are not recorded, while macro striations do appear over the surface of larger crystals. The recorded Sq mean value is 0.1 μm, and the mean Sv value is 0.3 μm (Supplementary Table 5).

Fresh hide cleaning with ochre left ochre-rich compounds characterized by a very distinct patchy distribution and a very smooth and compact appearance (Fig. 2d). Overall, at the edges of the compact patches, the residue has a powdery appearance. Patch-free areas are also visible with quartz grains covered by grease-rich film and collagen fibres.

The surface microrelief appears rounded. The crystal grains are amalgamated, and their surfaces abraded (Fig. 2e,f). When visible, the edges of the grains appear highly rounded. Pits and striations are not recorded. The recorded Sq values resulted in a mean of 0.05 μm, while the mean Sv value is 0.18 μm (Supplementary Table 5).

Dry bone polishing produced a white powdery residue well dispersed across the entire surface. Within the powder, we noticed the regular formation of glossy yellowish spots with a translucent and cracked appearance. Such glossy spots can also be densely striated (Fig. 2g). After use, the surface is characterized by a levelled microrelief. In some cases, voids generated by the detachment of crystal grains are detected. Polishing of dry bone affected mostly the surfaces of the grains, which appear lightly abraded (Fig. 2h,i). The edges of the grains are generally sharp, yet the contiguous appearance limits their visibility. Striations and pits are not visible. A mean Sq value of 0.04 μm is recorded across the surface, along with a mean Sv value of 0.15 μm (Supplementary Table 5).

Tendon processing produced a white powdery residue filling the surface voids with a spot-like distribution resembling vegetal material. Such patches of residues appear yellowish in colour when observed at higher magnification and superimposed collagen structures are also evident in the compressed residual matrix (Fig. 2j). At 50× of magnification, the utilized surface appears irregular, characterized by frequent voids due to the mechanical extraction of grains occurring during the use. The surfaces of the grains are crushed and fractured, while the edges are mostly sharp (Fig. 2k,l). Neither pits nor striations are present. Across the surface, a mean Sq value of 0.03 μm is recorded, while the mean recorded Sv value is 0.14 μm (Supplementary Table 5).

**Figure 3:**
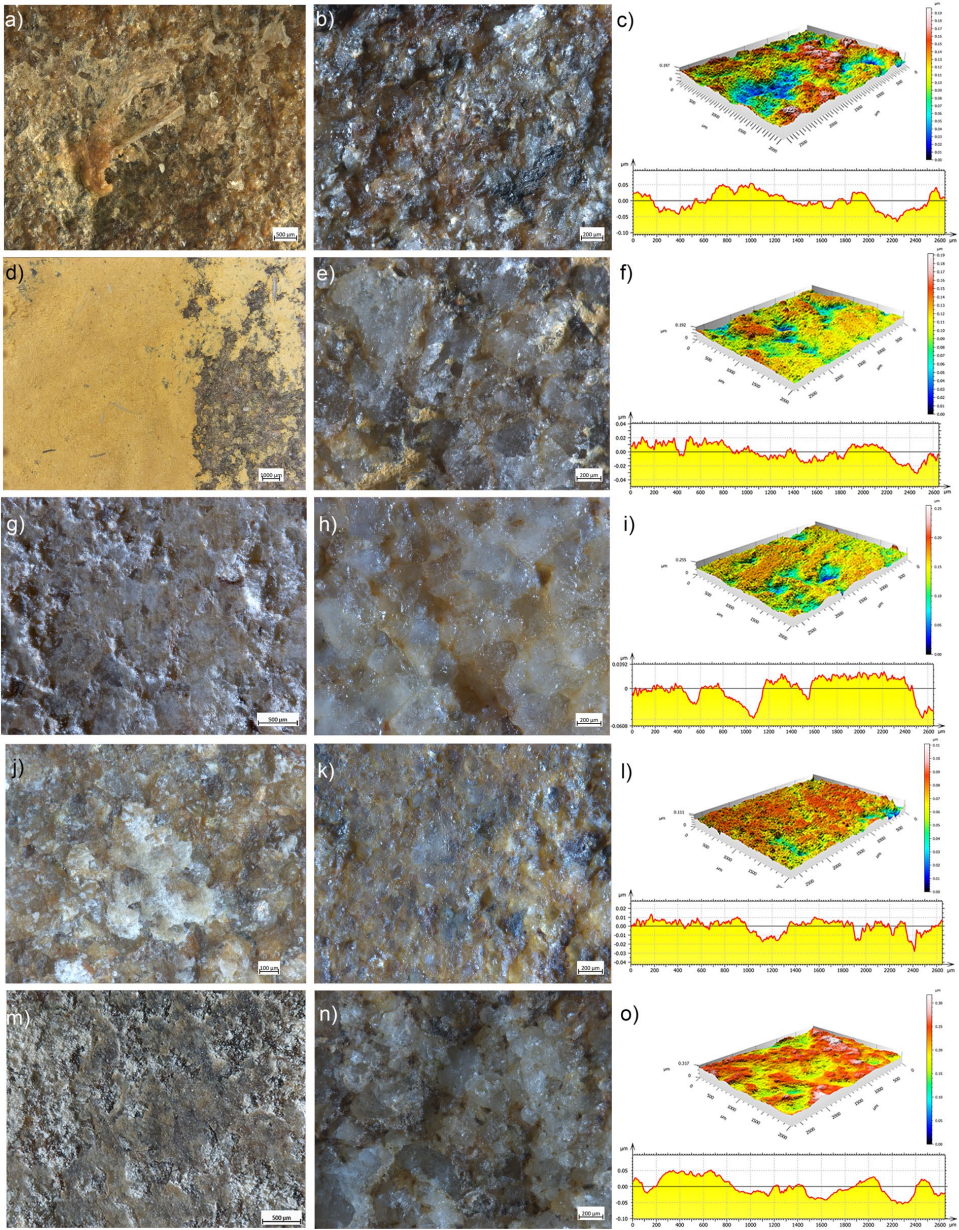
Use wear and residues observed on the experimental GST surfaces. (**a**) residues deriving from splitting metapodials; (**b**) use wear observed at 50×; (**c**) 3D rendering and profile of an area of the utilized surface; (**d**) residues deriving from mixing ochre with marrow; (**e**) use wear observed at 50×; (**f**) 3D rendering and profile of an area of the utilized surface; (**g**) residues deriving from grinding oat; (**h**) use wear observed at 50×; (**i**) 3D rendering and profile of an area of the utilized surface; (**j**) residues deriving from grinding wild grass grains; (**k**) use wear observed at 50×; (**l**) 3D rendering and profile of an area of the utilized surface; (**m**) residues deriving from crushing and grinding acorns; (**n**) use wear observed at 50×; (**o**) 3D rendering and profile of an area of the utilized surface.

Splitting metapodials left very greasy surfaces with patches of residue primarily distributed along the edges and across the central area of the stone base. Residues consist in compressed periosteum fibres mixed with fat as well as isolated collagen fibres. Across the central area of the tool, a highly compressed amorphous fat-rich yellowish compound is distributed in patches characterized by mud-cracked appearance and isolated collagen fibres (Fig. 3a). At 50× of magnification, the microrelief of the surface appears rounded, with the crystal grains well distinguishable one from another. The surface of the grains is abraded and, in some cases, characterized by microfractures, while the edges are characterized by a low degree of rounding (Fig. 3b,c). Neither pits nor striations are present. The measure of the Sq and Sv parameters returned mean values of 0.04 μm and 0.14 μm, respectively (Supplementary Table 5).

#### Organic and inorganic matters (marrow mixed with ochre)

Mixing marrow and ochre produced a compact and continuous film, which also entrapped collagen fibres (Fig. 3d).

At 50× of magnification, the microrelief of the utilized surface appears levelled. The crystal grains exhibit a contiguous appearance and are modified both in their edges and surface (Fig. 3e,f). The edges of the grains are highly rounded, and striations are visible on top of the grains. Pits are not visible. The recorded mean Sq value is 0.02 μm, and the mean Sv value is 0.09 μm (Supplementary Table 6).

#### Vegetal matters

Oat grinding left a smooth layer adhering the stone surface connecting the crystal grains into a flat, sometimes striated, surface, barely distinguishable from the stone itself (Fig. 3g). Such a layer is not always continuous, and depressions appear at low magnification. A powdery residue rich in starch and other vegetal structures fills the voids in the less used areas of the tool. At 50× of magnification, the surface microrelief is levelled (Fig. 3h,i). The crystal grains appear contiguous with abraded surfaces, and the edges are highly rounded. Striations are sometimes visible across the grain surfaces. The mean Sq value is 0.02 μm, while the mean Sv value is 0.16 μm (Supplementary Table 7).

Wild grass grains processing produced a translucent film, which covers and connects the crystal grains. Within such film, distinct yellowish, and striated patches alternate with a powdery residue rich in starch and other vegetal structures (Fig. 3j). The microrelief of the utilized surface appears levelled. The crystal grains exhibit an amalgamate appearance, and their surfaces are abraded. In most of the cases, the edges of the grains are not visible apart for isolated instances in which they appear highly rounded (Fig. 3k,l). Pits are not visible, while seldom striations are present. Across the surface, the mean Sq value is 0.02 μm, and the mean Sv value is 0.09 μm (Supplementary Table 7).

Acorn grinding produced a white powdery residue filling the surface voids with a spot-like distribution and brownish vegetal structures visible in the matrix. Such areas of powdery residues are dispersed on the surface in combination with greasy brownish patches, appearing like rough crusts adhering to the powdery residue on the stone surface (Fig. 3m). Likely, such crust is formed by the compression and friction exerted on top of the powdery residue during the functional activity. At 50× of magnification, the surface microrelief appears irregular. The grain appearance is contiguous, and, in most cases, each single grain is distinguishable (Fig. 3n,o). The surfaces of the crystals are characterized by abrasions and fractures, and their edges are rounded. Neither pits nor striations are visible. The mean Sq value is of 0.04 μm along with a mean Sv of 0.17 μm (Supplementary Table 7).

### The role of residue in surface quantification

**Figure 4:**
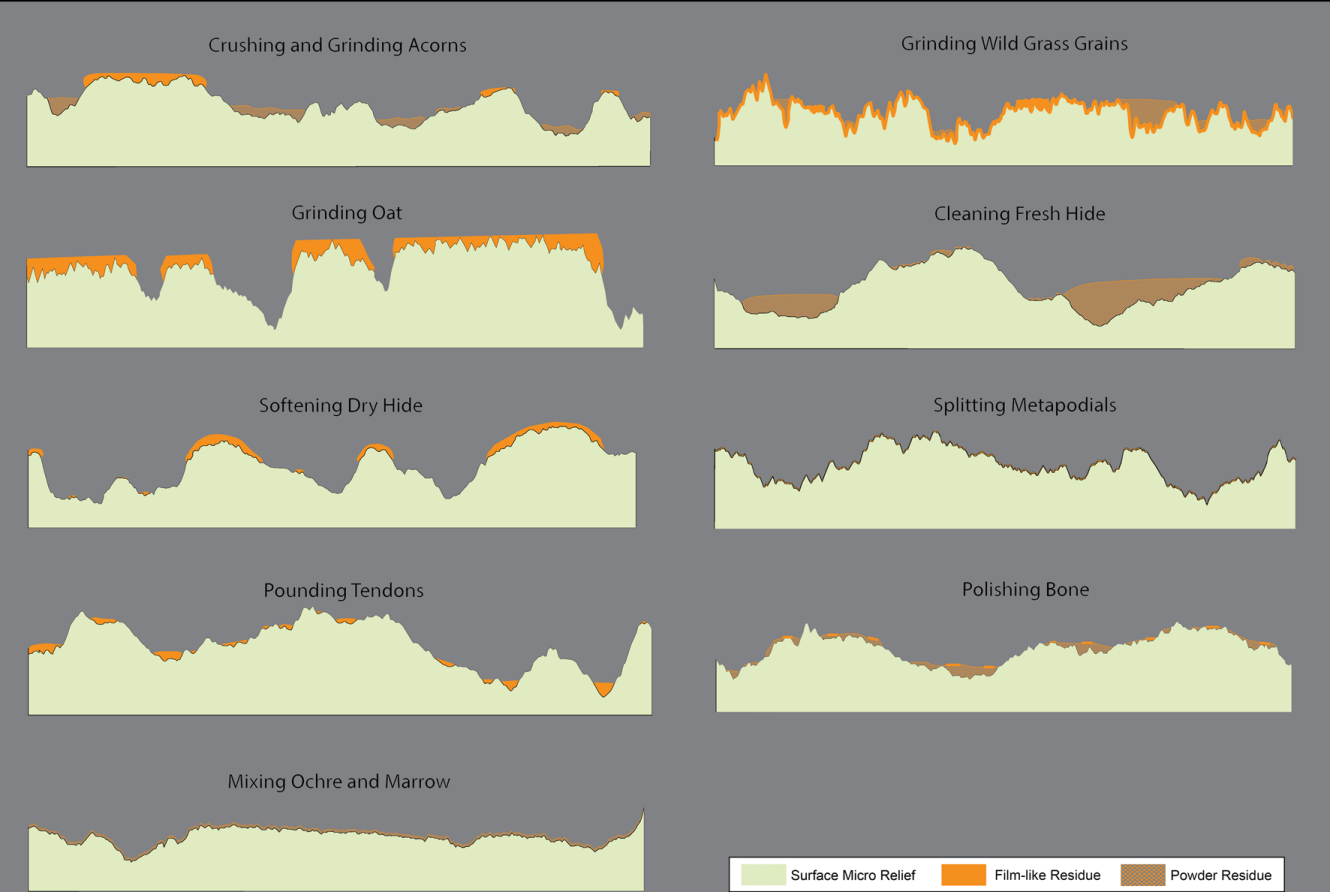
Observed disposition of residues according to the different activities performed and heir relationship with the surface micro-relief.

**Figure 5:**
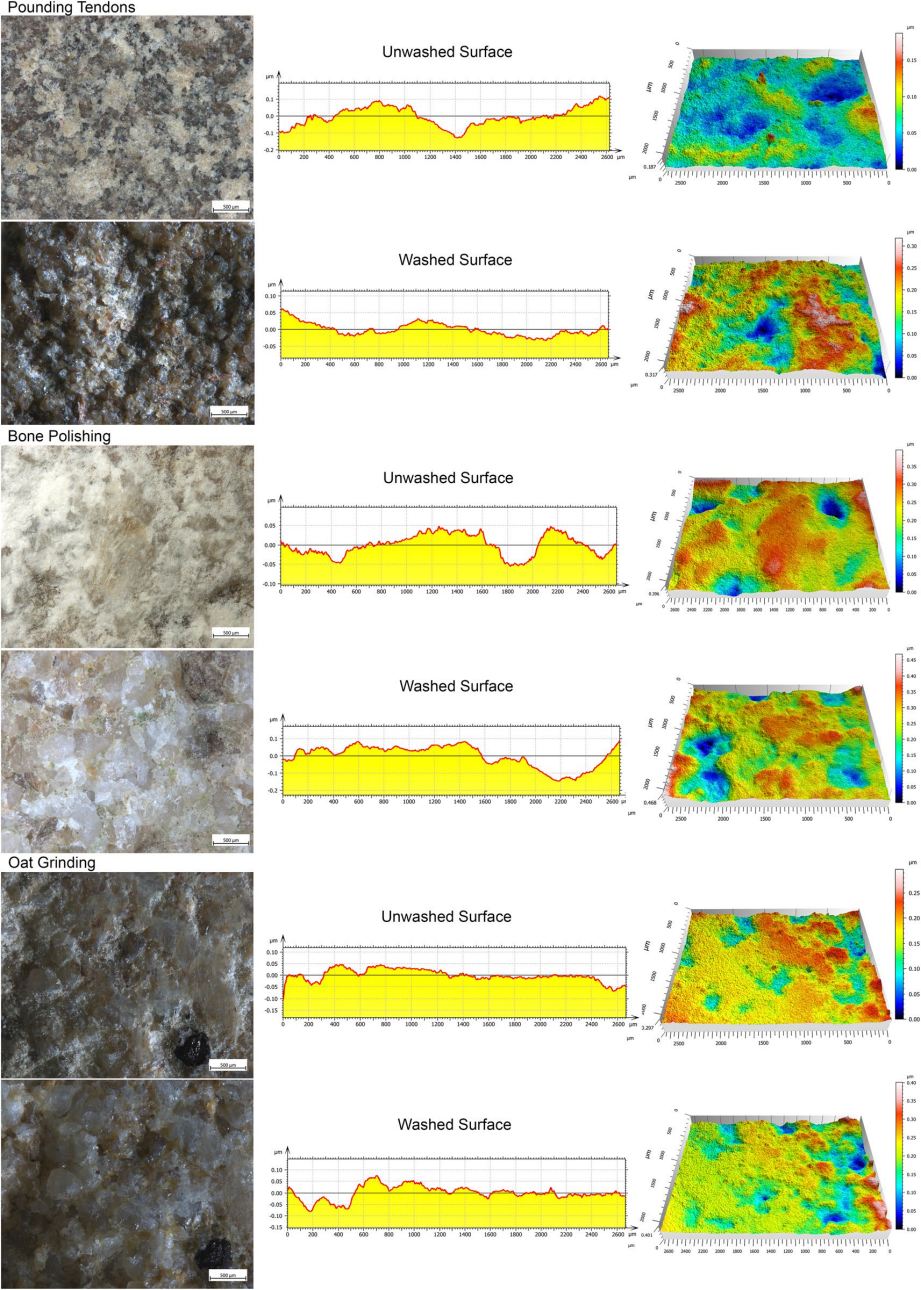
Comparison of the topography of unwashed and washed surfaces related to the processing of tendons, bone and oat.

During our experimental framework, we noticed relevant differences in the appearance of the utilized surfaces before and after the washing procedure. In particular, the disposition and appearance of residues seem to be strictly related to the surface profile resulting from the gesture performed (Fig. 4). Variations in the micro-relief are visible both qualitatively and quantitatively (Supplementary Table 8). We noticed that significant changes in the surface microrelief are connected with the development of three different types of residual matter: (a) powder, (b) compressed patches, and (c) film adhering over the used surface. Such variations of topography are particularly evident on the tools used to process oat, bone and tendons. On the tool used for oat processing, the residue covers the highest surface areas flattened by use, leading to the development of a continuous, homogenous crust, frequently characterized by linear features. Such adhering layers are very embedded in the stone matrix and sometimes hard to distinguish from the actual stone surface. Once such film is washed away, the surface topography changes becoming rougher with an increase in the recorded Sq (+ 0.019 μm) and Sv values (+ 0.02 μm) (Fig. 5).

In the case of tendon pounding, patches of residues filled such voids produced by grains removal leading to an overall smooth surface with a regular topography. As observed in the case of oat processing, once washed, the stone surface appears rougher, and the actual depth of the depression is revealed (Sq + 0.029 μm; Sv + 0.1 μm) (Fig. 5).

Concerning bone polishing, a powdery residue is spread across the surface and frequently covered by glossy striated spots. Once washed, striations are not visible anymore, and the used surface microrelief is slightly smoother compared to the one previously recorded (Sq + 0.008 μm; Sv + 0.019 μm) (Fig. 5).

## Discussions

In this paper we discussed the potential of a synergetic quantitative and qualitative approach applied to the functional study of experimental sandstone GSTs, emphasizing the contribution and the limits of quantitative surface morphometrics at a macro and micro-scale. A qualitative assessment of the use wear developed from the processing of animal, vegetal and mixed (animal and mineral) matters was coupled with the analysis of surface topography.

360° 3D surface morphometric analysis^11^ allowed for a complete topographic analysis of the tools’ surfaces measuring depression heights and roughness. It is important to state how these parameters (specifically surface depressions) and the patterns of modification they implicate, are strictly related to the raw material the tools are made of. In the case of sandstone, surface roughness was more informative than the depth of surface depressions, in particular for the identification of surface utilized areas. Specifically, the 3D 360° morphometric analysis allowed to recognize potential functional areas across the entire tool with a high degree of homogeneity (low roughness value) or heterogeneity (high roughness value). Two main spatial distribution patterns seem to characterize different material processing within our experimental assemblage: (a) clusters of low or high roughness surface areas, typical of tools employed for processing oat, ochre mixed with marrow and dry hide; and (b) diffused areas of low roughness surface characterizing the tools utilized for processing wild grass grains, acorns and fresh hide.

The three experimental replicas utilized as a base to split metapodials, pound tendons and polish dry bone costitute and exception to this pattern as the 360° 3D surface morphometric analysis could not allow the visual identification of the used surface areas. In particular, on these replicas the topography of the utilized portions returned similar values of roughness and/or surface depressions, thus impeding the quantitative recognition of the used areas. Accordingly, we believe the potential of 3D 360° morphometrics to identify functional areas on GSTs used in activities ending in a low degree of surface modification is limited.

**Figure 6:**
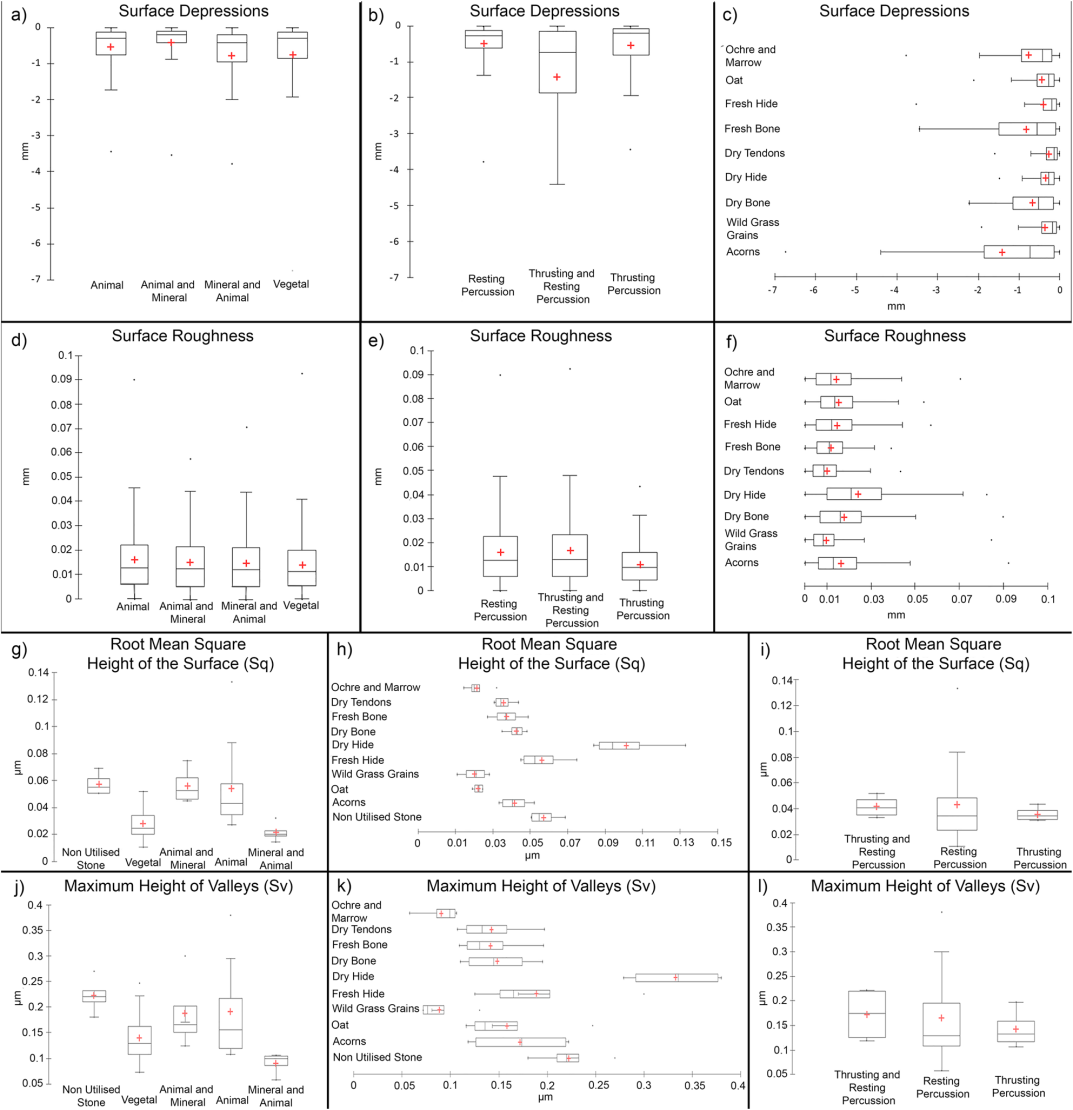
Boxplots of the measurements of surface depressions and surface roughness measured at a macro-scale through 3D 360° morphometric analysis (**a**–**f**) and of the measurements of root mean square height of the surface (Sq) and maximum height of valleys (Sv) recorded at 50× of magnification (**h**–**l**).

Moreover, while the 3D 360° surface morphometric analysis has potential for identifying potential utilized areas on sandstone GSTs, some limits can be met for detailed functional interpretations. Indeed, the measurement of depression heights and surface roughness of the used area of our experimental replicas are not discriminant for assessing the nature of the processed materials (e.g. animal, vegetal, animal and mineral or mineral and animal materials) (Fig. 6a–f). Only when surface depression and surface roughness values are expressed in terms of specific worked material, differences do emerge (Fig. 6c,d). In particular, crushing and grinding acorns resulted in the deepest surface depressions (mean − 1.4 mm; max. − 0.6 mm); the shallowest surface depressions are recorded across the surface of the tool utilized to grind wild grass grains (mean − 0.3 mm; max. − 1.9 mm); processing wild grass grains also resulted in the lowest values of surface roughness (mean 0.0098 mm; max. 0.084 mm); soften dry hide returned the highest roughness values (mean 0.024 mm). However, caution should be used as, at a macro-scale, these values can be affected by the raw material composition and the natural topographic features of the tools.

More consistent results were achieved through the analysis of surface topography at a micro-scale using higher magnifications (50×). Here, ISO 27158 parameters, such as the root mean square height of the surface (Sq), and the maximum height of valleys (Sv) provided useful functional information in terms of worked substances (Fig. 6g–k). The surface utilized to process mineral and animal matters (ochre mixed with marrow) resulted in the lowest Sq recorded (mean 0.022 μm). Animal and vegetal materials are clearly distinguishable according to the mean Sq values (0.054 μm and 0.028 μm, respectively). For these materials, a similar pattern is also recorded in terms of the maximum height of valleys (Sv), while mineral and animal materials returned the lowest measure (mean 0.09 μm). Vegetal materials are also characterized by shallow depressions (0.13 μm), while deeper voids were frequent across the surface utilized to process animal and mixed matters (animal and mineral), which resulted in similar mean heights (0.18 μm and 0.019 μm respectively). As in the case of the Sq measures, the used surfaces were characterized by Sv values differing from the ones recorded across a non-utilized sandstone surface (mean Sq 0.057 μm; mean Sv 0.22 μm).

When the Sq and Sv values of each worked substance were compared, the surface utilized to process oat and wild grass grains appeared to be the smoothest (Sq mean value 0.02 μm) among the replicas used to process vegetal matters. The tool utilized to soften dry hide was characterized by the highest Sq value recorded (mean 0.1 μm) within the animal matters. Concerning the height of surface depressions, the tool utilized for wild grass grains returned the lower Sv values (mean 0.089 μm), while the highest Sv measures were again associated to the surface utilized to soften dry hide (mean 0.33 μm).

While the measure of Sq and Sv appeared to be useful parameters for the interpretation of the worked matters, the same did not apply for the gesture performed. Indeed, no significant differences were observed for resting, thrusting, and mixed percussions (Fig. 6i,l). Similar Sq values characterized the three actions, and only small variations were recorded comparing the Sv measures. In this case, the deeper depressions were associated with a mixed motion of thrusting and resting percussion (mean Sv 0.17 μm), while shallower ones characterized the surfaces involved in thrusting or resting percussion (mean Sv 0.14 μm and 0.16 μm).

Overall, our results highlighted two main arguments against the exclusive application of quantitative analysis in the study of sandstone GSTs. As a matter of fact, qualitative variables associated with the description of macro and microwear provided a multitude of information that cannot be replaced with the application of surface measurements only. This is particularly evident when assessing of the gesture performed, as our data stressed the failure of quantitative analysis to provide useful information related to this matter. This proves it is essential to combine numerical data with the qualitative characteristics of use wear to pursue comprehensive functional interpretations.

Furthermore, we advise for the development of standard protocols in selecting specific used areas for surface quantification. In particular, our analysis revealed significant differences in the topographic measurements between washed and unwashed surface areas (Fig. 5). Similarly to what already observed for flint stone tools^31–33^, organic film may develop across the surface of GSTs due to different kind of mechanical and/or thermal stresses intervening during their use (see^28,34^). Sometimes, such adhering layers are so embedded in the stone matrix that the residue can erroneously be considered as the originally modified surface, thus leading to the quantification of topographic features unrelated to the used area. While understanding the nature and dynamics of the film development on GSTs will require the application of tribological approaches deriving from the field of material engineering, a trained eye for functional modifications on stone surfaces can recognize the presence of residues using standard optical microscopy and evaluate them accordingly^35^. In this regard, the researcher’s experience has an active role in improving the objective comprehension of the tool function^36^. However, also post-depositional alterations can affect the topography of used surfaces and influence their quantification^13^.

Limits do exist within the qualitative description of use wear as well, specifically concerning the degree of subjectivity in the assessment of the functional features. While generally not leading to incorrect interpretations, such bias narrows down the possibility of reproducing and comparing functional results amongst specialists. In this regard, the application of quantitative surface analyses should not be intended as a replacement of the well-established qualitative use wear observations but rather as a means for enriching the overall quality of the functional data.

Qualitative analyses have proven to be essential in establishing the accuracy of quantitative data. In particular, our results suggest that subjectivity characterizing qualitative use wear interpretation should not be intended as a methodological limit impeding the objective understanding of a tool’s function but rather as an interpretative advantage for enhancing the quality of the results obtained in quantitative analysis. In conclusion, we support the benefits of a synergetic functional approach on GST technology involving both the classic optical and quantitative analysis as a promising option for reliable interpretation of ancient tool use.

## Conclusion

In our work, we tested the application of a synergetic approach combining qualitative and quantitative analyses to the study of GSTs. Specifically, we coupled the observation and description of use wear at low magnification with the analysis of surface topography at a macro and micro-scales.

Through our study, we demonstrated how a 3D 360° morphometric analysis represents a useful tool to observe potentially utilized areas of GSTs. A limit of this method can be identified in its capacity to interpret the nature of the worked material or the gesture performed. Our analysis also suggested that the root mean square height of the surface (Sq), and the maximum height of valleys (Sv) are key parameters for identifying the worked material at higher magnification. However, as in the case of 3D 360° surface morphometric, also the values of Sq and Sv lack in providing a reliable interpretation of the performed gesture.

In conclusion, our results underline the importance of a synergetic approach to improve the quality and accuracy of GST functional interpretations and provide arguments against an exclusive application of quantitative approaches.

## Methods

The functional analysis presented in this paper builds upon two main phases:

1. An analysis of surface features performed at a macro-scale during which 3D models of the experimental replicas are processed through a 3D 360° surface morphometric analysis. In this way, a complete (360°) surface morphometric dataset is provided per tool and potential utilized areas of the tools are identified.
2. Analysis of the utilized areas of the experimental replicas performed using a digital stereomicroscope at 50× of magnifications, which allowed to:
  - quantify surface morphometric patterns following ISO 25718 standards.
  - identify and describe residues and surface modification patterns associated with each of the processed material and performed activities following the commonly utilized parameters applied in functional studies of GSTs^25,28,37^.

### 3D surface quantification

3D models of the experimental replicas were created through Close Range Photogrammetry^38^. Using the same acquisition standards applied by Zupancich et al.^12^ a total of 144 pictures (72 per side) were taken and processed using Agisoft Metashape v1.6.2

To analyze the topography of each tool in its entirety, a 360° surface analysis was performed following the methodology proposed by Benito-Calvo et al.^11^. At first, a convex hull was created in Meshlab (v.2020.03) for each tool. This consists of the smallest three-dimensional convex surface containing all the data of the 3D model and represents the reference from which the measure of surface elevation is calculated^39^.

3D meshes and their related convex hulls were then imported in the software Cloud Compare (v2.10.2)^40^ to measure the height of surface depressions and surface roughness, which represent the main surface characteristics of a GST that can be affected by use^25,28,37^. Surface depressions were calculated by measuring the distance occurring by the 3D mesh and its convex hull, while surface roughness was calculated using a 0.5 mm local neighbour radius. Along with the morphometric analysis of the entire stone tool, the utilized surfaces were extracted and compared. This allowed building a first dataset of morphometric measurements obtained at a macro-scale. Following this first step of the analysis, used areas were observed at low magnification (50×) using a digital stereo microscope. This provided a qualitative description of surface micro-relief, crystal grain modification patterns, presence or absence of pits and linear features associated with the use of the experimental replicas. Moreover, four surface areas of 2 × 2.5 mm were sampled across the used surfaces to perform surface measurements. ISO 25718 aerial surface parameters were utilized. Each surface sample was recorded at 50× of magnification using the same acquisition parameters (6 μm slices) and imported in the Mountains Map Premium (v.7.2) software from Digital Surf. Following Macdonald et al.^6^, each image was processed to remove non relevant data. The images were levelled, the form was removed using an F-Operator with a polynomial degree of 2, and a robust Gaussian filter (25 μm) was applied as well. Within the available range of ISO 25718 areal surface parameters, the root mean square height of the surface (*Sq*) and the maximum height of valleys (*Sv*) were considered. The selection of these two parameters was based upon their efficiency in the characterization and measure of surface topography^6,41,42^.

### Use wear and residue analysis

For the purposes of this research, the observation of use wear was performed only at low magnifications^43–46^. A Zeiss—Axio Zoom V16 Digital Stereoscope equipped with a PlanNeoFluar Z1x/0.25 FWD objective and 10× eyepieces, capable of magnifications ranging from 7× to 112×, was employed to identify the utilized areas of the tools. The modification patterns observed included micro-relief, grain morphology, pits and striations, described following well-established parameters commonly used in the functional study of GSTs^25,28,37,47,48^.

Before the microscopic observation of use wear, each of the experimental replicas was washed by hand using ultrapure water and a 2% neutral phosphate detergent solution (Derquim)^49^.

Residues were observed using a Zeiss—Axio Zoom V16 Digital Stereoscope equipped with a PlanNeoFluar Z1×/0.25 FWD objective and 10× eyepiece, capable of magnifications ranging from 7× to 112×. Residual materials adhering the surfaces were described according to their appearance, using variables such as morphology, texture, color and birefringency^50–52^. Potential features related to mechanical stresses affecting the appearance of the residues and related to use were also recorded. Identifiable morphological features of plant and/or animal structures were recorded along with their spatial distribution across the utilized surfaces. Both use wear and residues were documented using a Zeiss Axiocam 506 High Definition Colour Camera.

## Supplementary information

Supplementary file1

## Data Availability

All data generated or analyzed during this study are included in this published article (and its Supplementary Information files).
