# Supplementary material for: Functional analysis of sandstone ground stone tools: arguments for a qualitative and quantitative synergetic approach
Source: Sci Rep. 2020 Sep 25;10:15740. doi: 10.1038/s41598-020-72276-0 (PMC7519652; doi:10.1038/s41598-020-72276-0)
Supplement: Supplementary file 1 — Supplementary file1 [file 41598_2020_72276_MOESM1_ESM.pdf]

Functional Analysis of Sandstone Ground Stone Tools: Arguments for a Qualitative and Quantitative Synergetic Approach

Andrea Zupancich[1**] and Emanuela Cristiani[1*]

[1]DANTE Diet and Ancient Technology Laboratory – Department of Oral and Maxillo Facial Sciences, Sapienza University of Rome
Via Caserta 6, 00161 Rome (Italy)

[*]Corresponding author: Emanuela Cristiani
[**]co-corresponding author: Andrea Zupancich

Contact information for corresponding and co-corresponding authors:
emanuela.cristiani@uniroma1.it
andrea.zupancich@uniroma1.it

ID	SrM_8b	SrM_8	SrM_13	SrM_17	SrM_16	SrM_6	SrM_9	SrM_7	SrM_5
**Raw Material**	Sandstone	Sandstone	Sandstone	Sandstone	Sandstone	Sandstone	Sandstone	Sandstone	Sandstone
**Raw Material Origin**	Bojetinska River, Serbia	Bojetinska River, Serbia	Bojetinska River, Serbia	Bojetinska River, Serbia	Bojetinska River, Serbia	Bojetinska River, Serbia	Bojetinska River, Serbia	Bojetinska River, Serbia	Bojetinska River, Serbia
**Role**	Passive Base	Passive Base	Passive Base	Passive Base	Passive Base	Active Tool	Active Tool	Passive Base	Passive Base
**Morphology**	Subangular	Subangular	Subangular	Subangular	Subangular	Subangular	Subangular	Subangular	Subangular
**Length (mm)**	133	133	126	143	225	118	115	119,8	140
**Width (mm)**	114	114	105	119	201	91	80	100	94
**Thickness (mm)**	53	53	168	77	48,2	66	40	88,6	81,8
**Weight (g)**	1450	1450	1510	2258	3167	958	515	1706	1815
**Nature of Worked Material**	Animal	Vegetal	Animal	Vegetal	Vegetal	Animal	Animal	Animal	Mineral
**Worked Material**	Dry Tendons	Aegilops	Bone	Oat (Avena sp)	Acorn	Drying Hide	Fresh hide	Metapodial bones	Ochre
**Additive**	No	No	No	No	No	Ochre	Ochre	No	Marrow
**Activity**	Pounding	Back and Forth	Back and Forth	Grinding	Pounding and Grinding	Softening Dry Hide	Cleaning Fresh Hide with Ochre	Splitting the Bone to Access Marrow Through Indirect	Mixing Ochre with Animal Matter (Marrow)
**Gesture**	Thrusting Percussion	Resting Percussion	Resting Percussion	Resting Percussion	Thrusting and Resting Percussion	Resting Percussion	Resting Percussion	Thrusting Percussion	Resting Percussion
**Time of Use(min)**	30	180	25	180	180	60	45	60	35

SI Table 1. Details of the GST replicas and of the experimental activities performed.

Activity		Entire Tool		Utilised Area	
Splitting Metapodials		Surface Depressions Depth (mm)	Surface Roughness (mm)	Surface Depressions Depth (mm)	Surface Roughness (mm)
	Min.	0	0	0	0
	Max.	-9	0.12	-3	0.085
	Median	-0.4	0.011	-0.05	0.013
	Mean	-0.9	0.013	-0.09	0.015
	StD	0.001	0.000010	0.0009	0.00001
Cleaning Fresh Hide with Ochre		Surface Depressions Depth (mm)	Surface Roughness (mm)	Surface Depressions Depth (mm)	Surface Roughness (mm)
	Min.	0	0	0	0
	Max.	-3	0.21	-3	0.18
	Median	-0.02	0.015	-0.02	0.013
	Mean	-0.04	0.018	-0.05	0.015
	StD	0.0005	0.000015	0.0007	0.000012
Softening Dry Hide		Surface Depressions Depth (mm)	Surface Roughness (mm)	Surface Depressions Depth (mm)	Surface Roughness (mm)
	Min.	0	0	0	0
	Max.	-7	0.29	-3	0.14
	Median	-0.3	0.017	-0.3	0.019
	Mean	-0.5	0.021	-0.4	0.023
	StD	0.0007	0.000017	0.0003	0.000017
Abrading Bone		Surface Depressions Depth (mm)	Surface Roughness (mm)	Surface Depressions Depth (mm)	Surface Roughness (mm)
	Min.	0	0	0	0
	Max.	-10	0.23	-2	0.12
	Median	-0.2	0.016	-0.2	0.015
	Mean	-0.8	0.018	-0.4	0.018
	StD	0.001	0.000014	0.0004	0.000013
Pounding Tendons		Surface Depressions Depth (mm)	Surface Roughness (mm)	Surface Depressions Depth (mm)	Surface Roughness (mm)
	Min.	0	0	0	0
	Max.	-3	0.1	-0.1	0.04
	Median	-0.1	0.01	-0.14	0.08
	Mean	-0.2	0.012	-0.2	0.01
	StD	0.0003	0.0009	0.0003	0.000008

SI Table 2. Summary statistics of the surface depression height and surface roughness recorded on the entire tools and their associated functional areas utilised to process animal matters.

Activity		Entire Tool		Utilised Area	
Crushing and Grinding Acorns		Surface Depressions Depth (mm)	Surface Roughness (mm)	Surface Depressions Depth (mm)	Surface Roughness (mm)
	Min.	0	0	0	0
	Max.	-12	0.2	-7	0.15
	Median	-0.5	0.06	-1.1	0.013
	Mean	-1.4	0.09	-1.5	0.016
	StD	0.0018	0.000008	0.0014	0.000013
Grinding Oat		Surface Depressions Depth (mm)	Surface Roughness (mm)	Surface Depressions Depth (mm)	Surface Roughness (mm)
	Min.	0	0	0	0
	Max.	-6.5	0.19	-6	0.13
	Median	-0.3	0.015	-0.5	0.013
	Mean	-0.6	0.017	-1	0.015
	StD	0.0008	0.000013	0.0013	0.000012
Grinding Aegilops		Surface Depressions Depth (mm)	Surface Roughness (mm)	Surface Depressions Depth (mm)	Surface Roughness (mm)
	Min.	0	0	0	0
	Max.	-3.7	0.1	-2	0.9
	Median	-0.1	0.01	-0.2	0.09
	Mean	-0.2	0.012	-0.4	0.011
	StD	0.00035	0.000009	0.0004	0.000009

SI Table 3. Summary statistics of the surface depression height and surface roughness recorded on the entire tools and their associated functional areas utilised to process vegetal matters.

Activity		Entire Tool		Utilised Area	
Mixing Ochre and Marrow		Surface Depressions Depth (mm)	Surface Roughness (mm)	Surface Depressions Depth (mm)	Surface Roughness (mm)
	Min.	0	0	0	0
	Max.	-4.9	0.18	-3.9	0.09
	Median	-0.3	0.016	-0.4	0.013
	Mean	-0.4	0.019	-0.7	0.016
	StD	0.00055	0.000014	0.0008	0.000012

SI Table 4. Summary statistics of the surface depression height and surface roughness recorded on the entire tools and their associated functional areas utilised to process inorganic mineral and stone matters

	Statistic	Sq (µm)	Sv (µm)
Splitting Metapodilas	Minimum (µm)	0.027	0.109
	Maximum (µm)	0.049	0.196
	Median (µm)	0.037	0.130
	Mean (µm)	0.038	0.141
	Standard deviation (n)	0.008	0.034
Cleaning Fresh Hide with Ochre	Minimum (µm)	0.045	0.125
	Maximum (µm)	0.075	0.3
	Median (µm)	0.053	0.165
	Mean (µm)	0.056	0.189
	Standard deviation (n)	0.012	0.066
Softening Dry Hide	Minimum (µm)	0.084	0.279
	Maximum (µm)	0.133	0.38
	Median (µm)	0.094	0.336
	Mean (µm)	0.101	0.333
	Standard deviation (n)	0.019	0.045
Abrading Bone	Minimum (µm)	0.035	0.11
	Maximum (µm)	0.052	0.195
	Range	0.019	0.085
	Median (µm)	0.041	0.145
	Mean (µm)	0.042	0.149
	Standard deviation (n)	0.005	0.034
Pounding Tendons	Minimum (µm)	0.03	0.10
	Maximum (µm)	0.048	0.19
	Median (µm)	0.034	0.13
	Mean (µm)	0.035	0.14
	Standard deviation (n)	0.005	0.034

SI Table 5. Summary statistics for the surface measurements obtained at 50x of magnification of the GSTs utilised to process animal matters. Sq (Root mean square height of the surface); Sv (maximum height of valleys).

	Statistic	Sq (µm)	Sv (µm)
Crushing and Grinding Acorns	Minimum	0.033	0.118
	Maximum	0.052	0.222
	Median	0.041	0.174
	Mean	0.042	0.172
	Standard deviation (n)	0.007	0.004
Grinding Oat	Minimum	0.019	0.116
	Maximum	0.025	0.247
	Median	0.023	0.136
	Mean	0.022	0.159
	Standard deviation (n)	0.002	0.052
Grinding Aegilops	Minimum	0.011	0.072
	Maximum	0.028	0.130
	Median	0.021	0.077
	Mean	0.020	0.089
	Standard deviation (n)	0.007	0.024

SI Table 6. Summary statistics for the surface measurements obtained at 50x of magnification of the GSTs utilised to process vegetal matters. Sq (Root mean square height of the surface); Sv (maximum height of valleys).

	Statistic	Sq (µm)	Sv (µm)
Mixing Ochre with Marrow	Minimum	0.014	0.058
	Maximum	0.032	0.106
	Median	0.02	0.1
	Mean	0.022	0.091
	Standard deviation (n)	0.006	0.019

SI Table 7. Summary statistics for the surface measurements obtained at 50x of magnification of the GST utilised to process inorganic matters. Sq (Root mean square height of the surface); Sv (maximum height of valleys).

	Unwashed Surface		Washed Surface		Variation	
	Sq (µm)	Sv (µm)	Sq (µm)	Sv (µm)	Sq (µm)	Sv (µm)
Pounding Tendons	0.020	0.06	0.049	0.17	0.029	0.11
Polishing Bone	0.056	0.22	0.065	0.24	0.009	0.02
Grinding Oat	0.027	0.17	0.046	0.19	0.019	0.02

SI Table 8. Comparison between the root mean square height of the surface (Sq) and the maximum height of valleys (Sv) recorded on the same area of the tool before and after washing.
